# Supplementary figures and images for: RecombineX: A generalized computational framework for automatic high-throughput gamete genotyping and tetrad-based recombination analysis
Source: PLoS Genet. 2022 May 9;18(5):e1010047. doi: 10.1371/journal.pgen.1010047 (PMC9119626; doi:10.1371/journal.pgen.1010047)

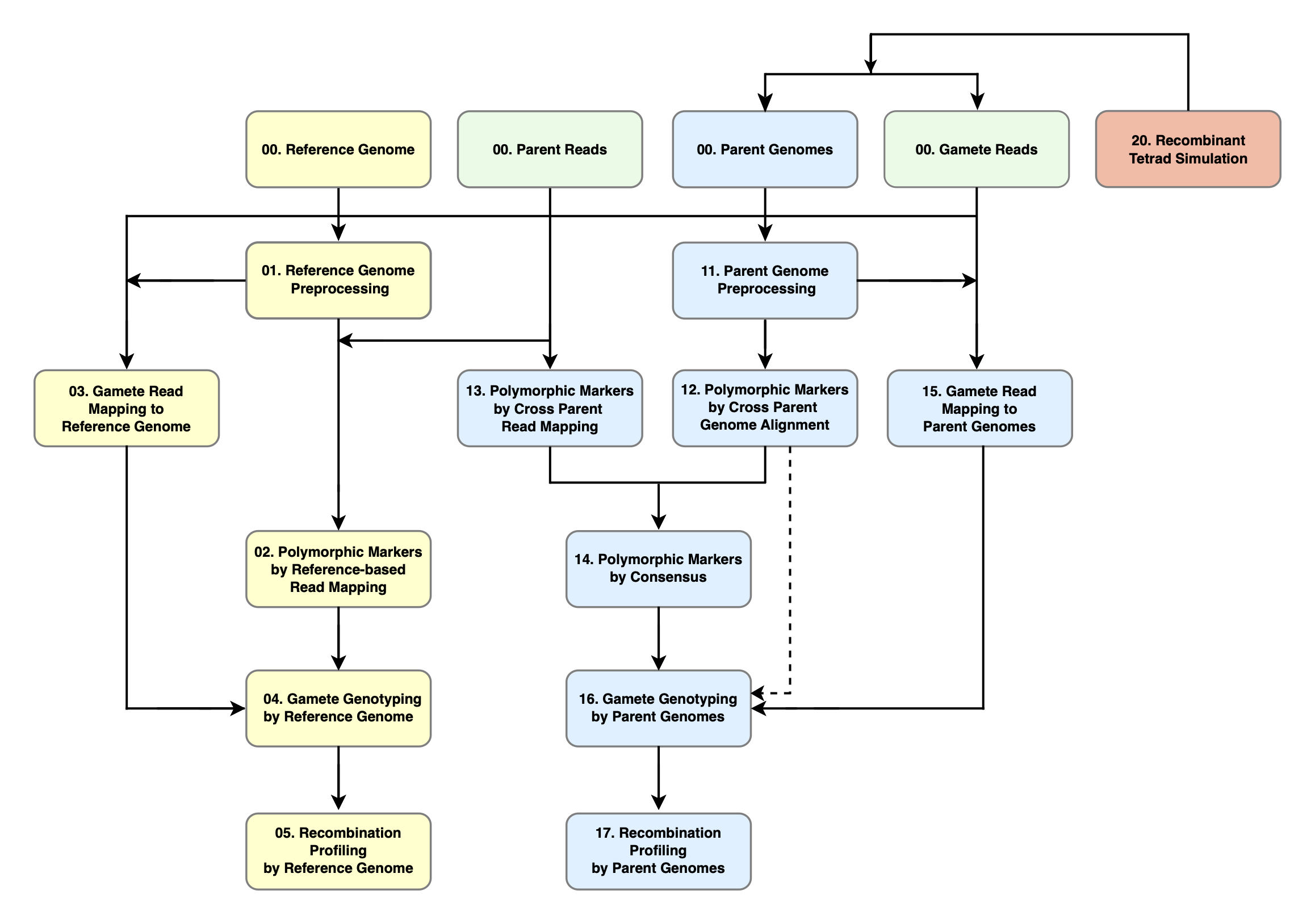

Supplement: S1 Fig — RecombineX consists of seventeen task-specific modules, with six modules dedicated for the reference-based mode (colored in yellow) and eight modules dedicated for the parent-based mode (colored in blue). As for the three remaining modules, two are designed for both reference-based and parent-based modes (colored in green), with the last one for simulation analysis (colored in red). (TIF) [file pgen.1010047.s001.tif]

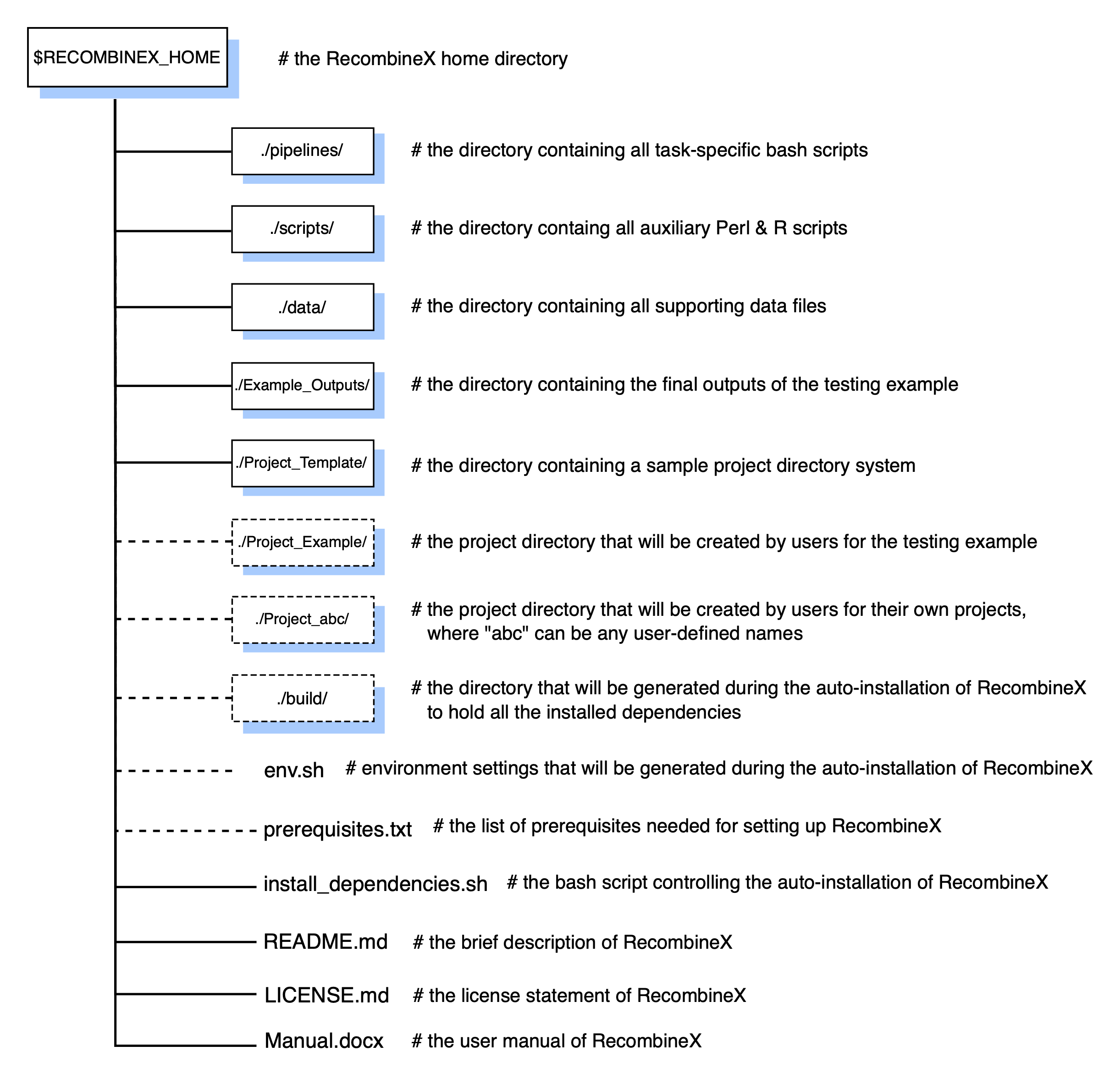

Supplement: S2 Fig — The pre-shipped top-level directories and individual files of RecombineX are denoted with solid lines. Additional directories and files to be generated during the installation of RecombineX are denoted with dashed lines. (TIF) [file pgen.1010047.s002.tif]

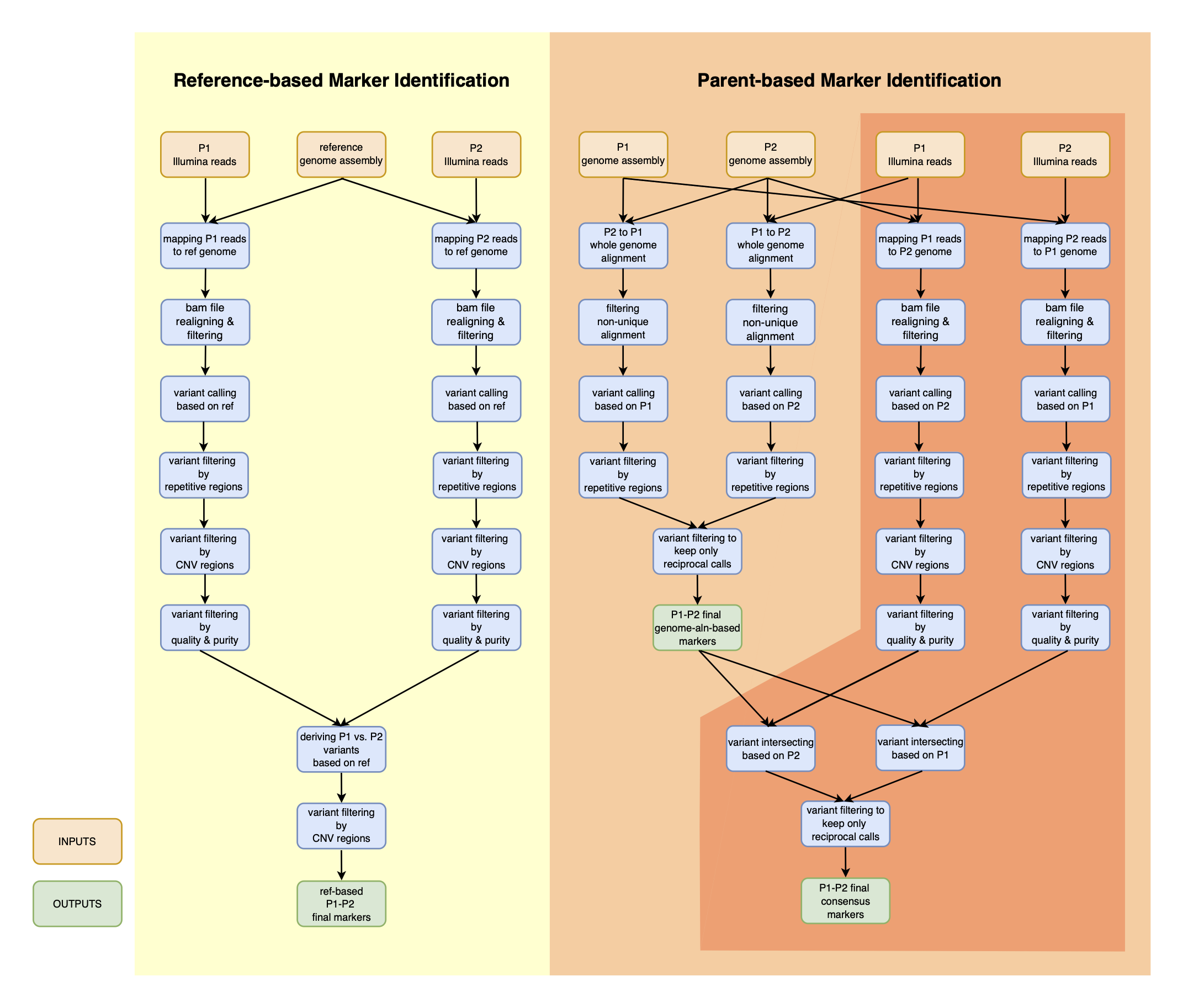

Supplement: S3 Fig — Two marker identification modes are supported: the reference-based mode (colored in yellow) and the parent-based mode (colored in orange). (TIF) [file pgen.1010047.s003.tif]

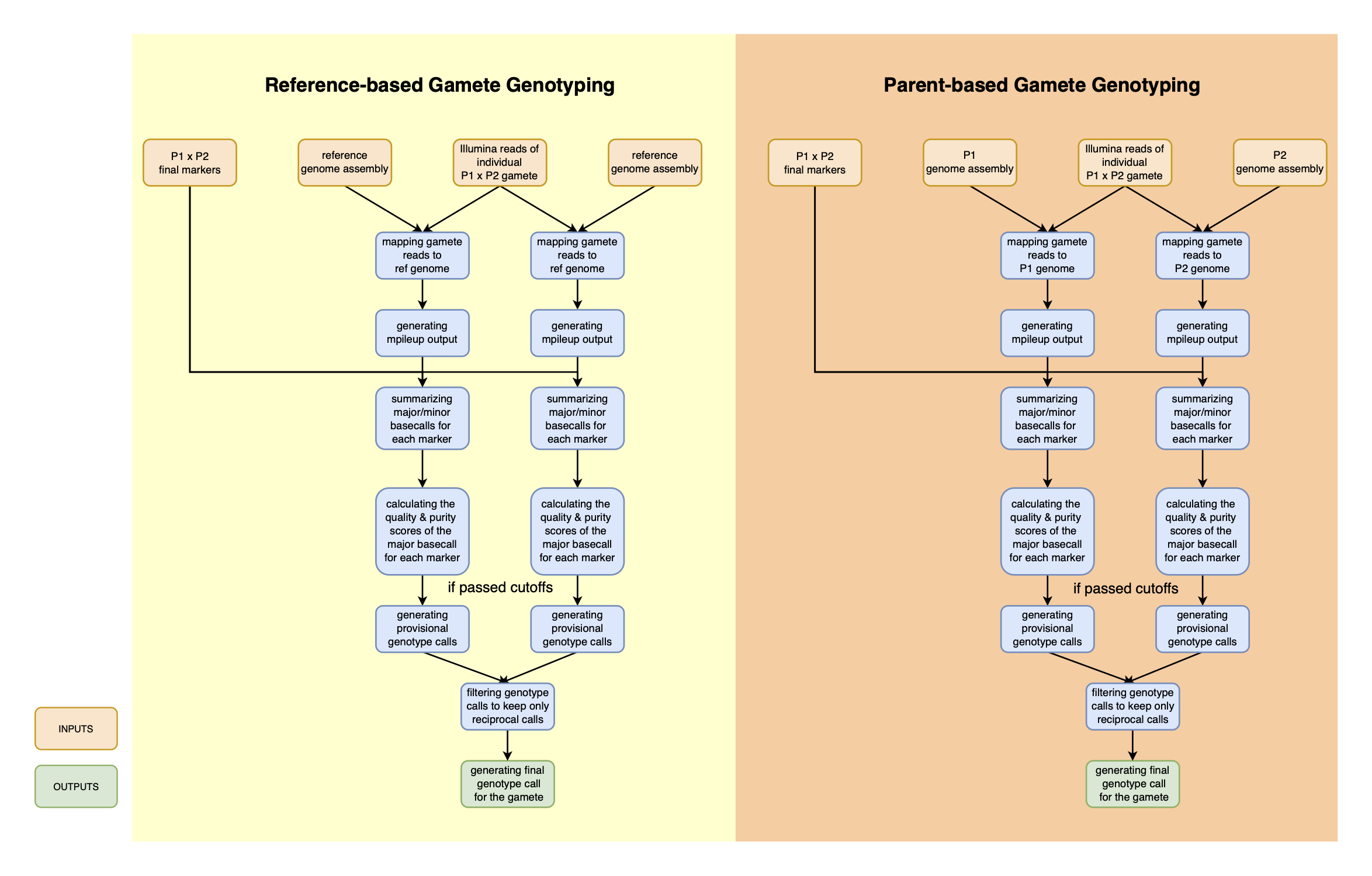

Supplement: S4 Fig — Two genotyping modes are supported: the reference-based mode (colored in yellow) and the parent-based mode (colored in orange). (TIF) [file pgen.1010047.s004.tif]

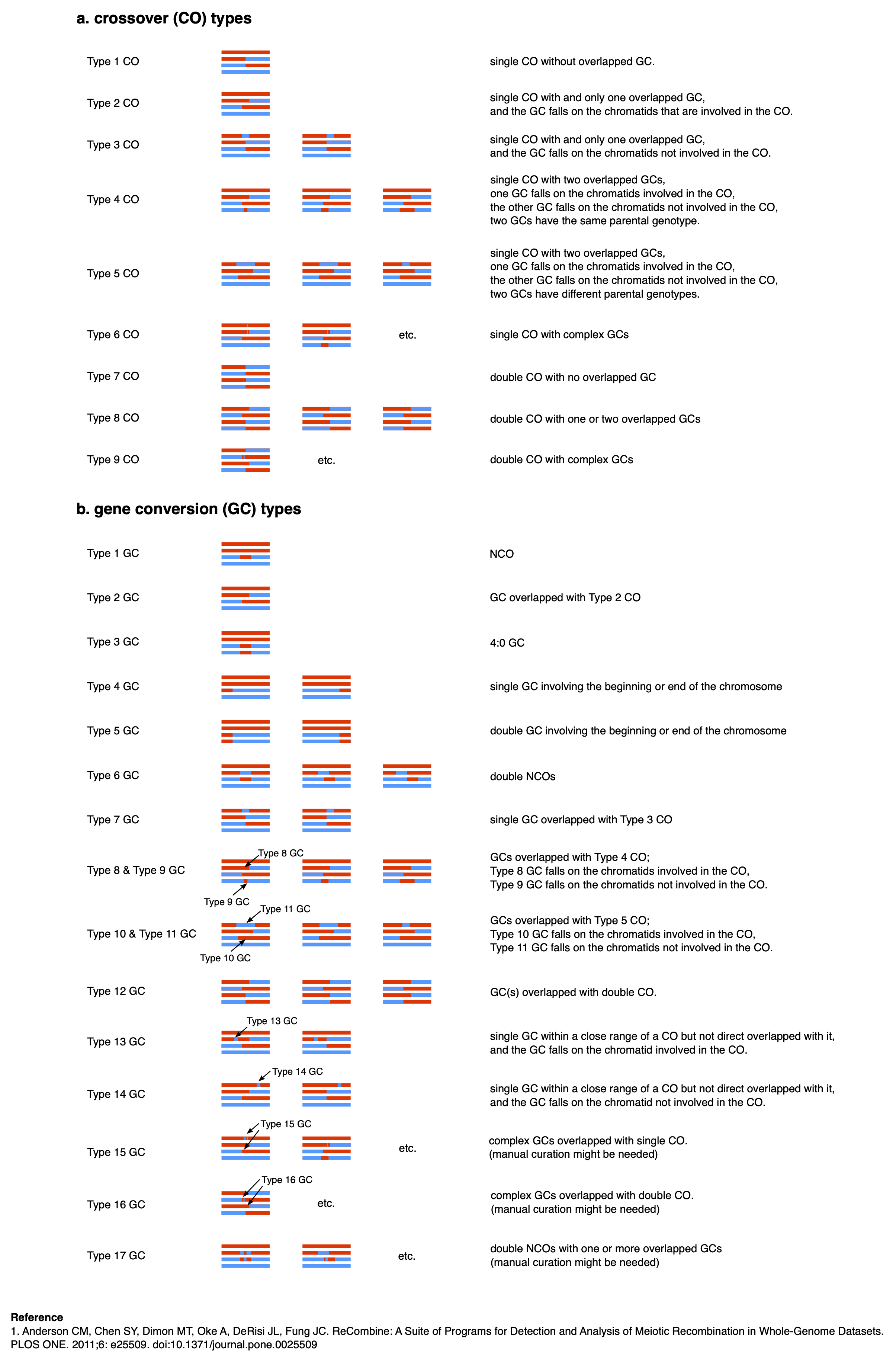

Supplement: S5 Fig — The definition and example of different CO and GC types are shown in panel a and panel b respectively. This classification scheme is designed based on the original ReCombine recombination event classification scheme [19] with additional modifications. (TIF) [file pgen.1010047.s005.tif]

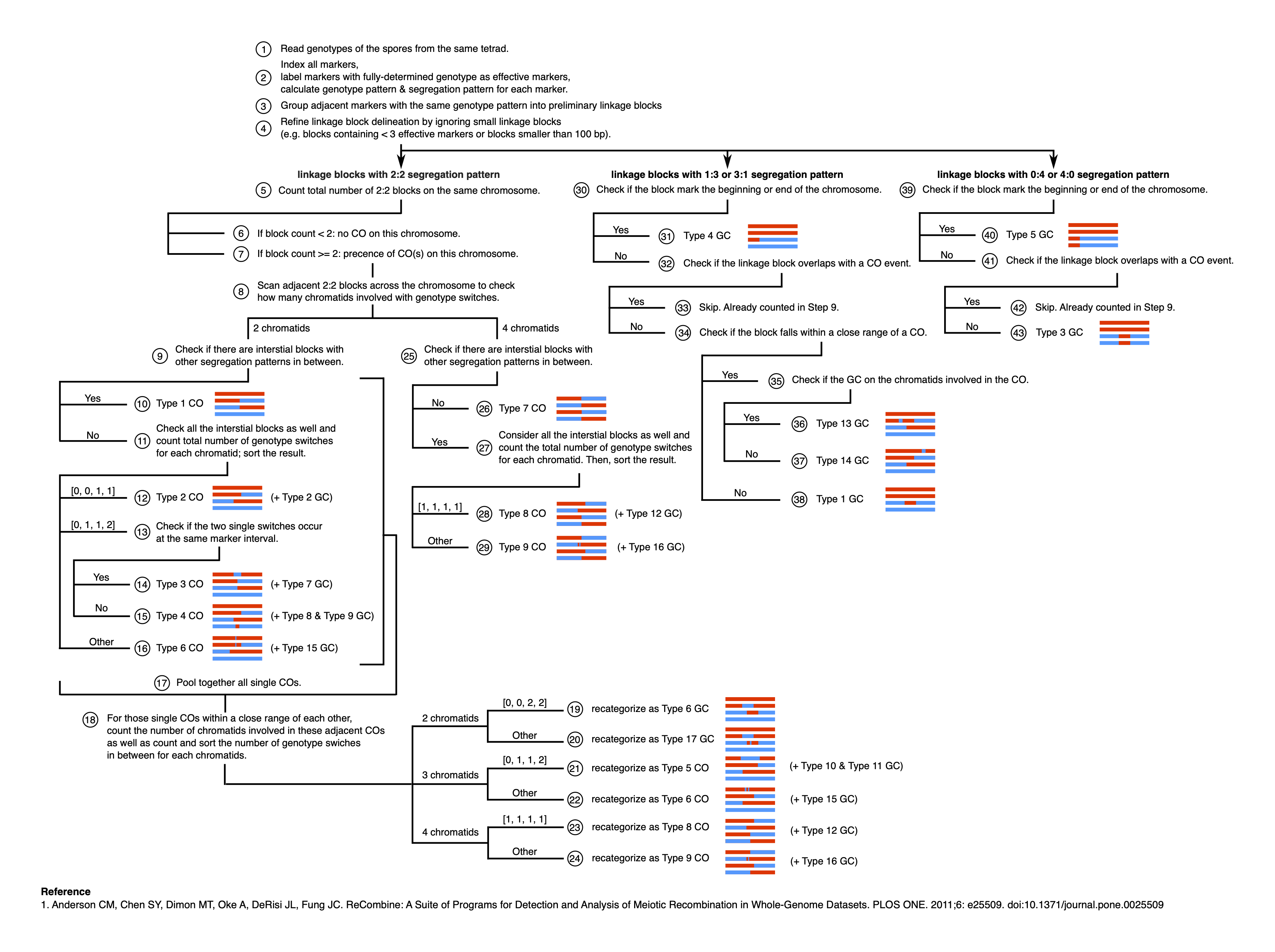

Supplement: S6 Fig — This algorithm is designed based on the original ReCombine algorithm [19] with additional modifications. (TIF) [file pgen.1010047.s006.tif]

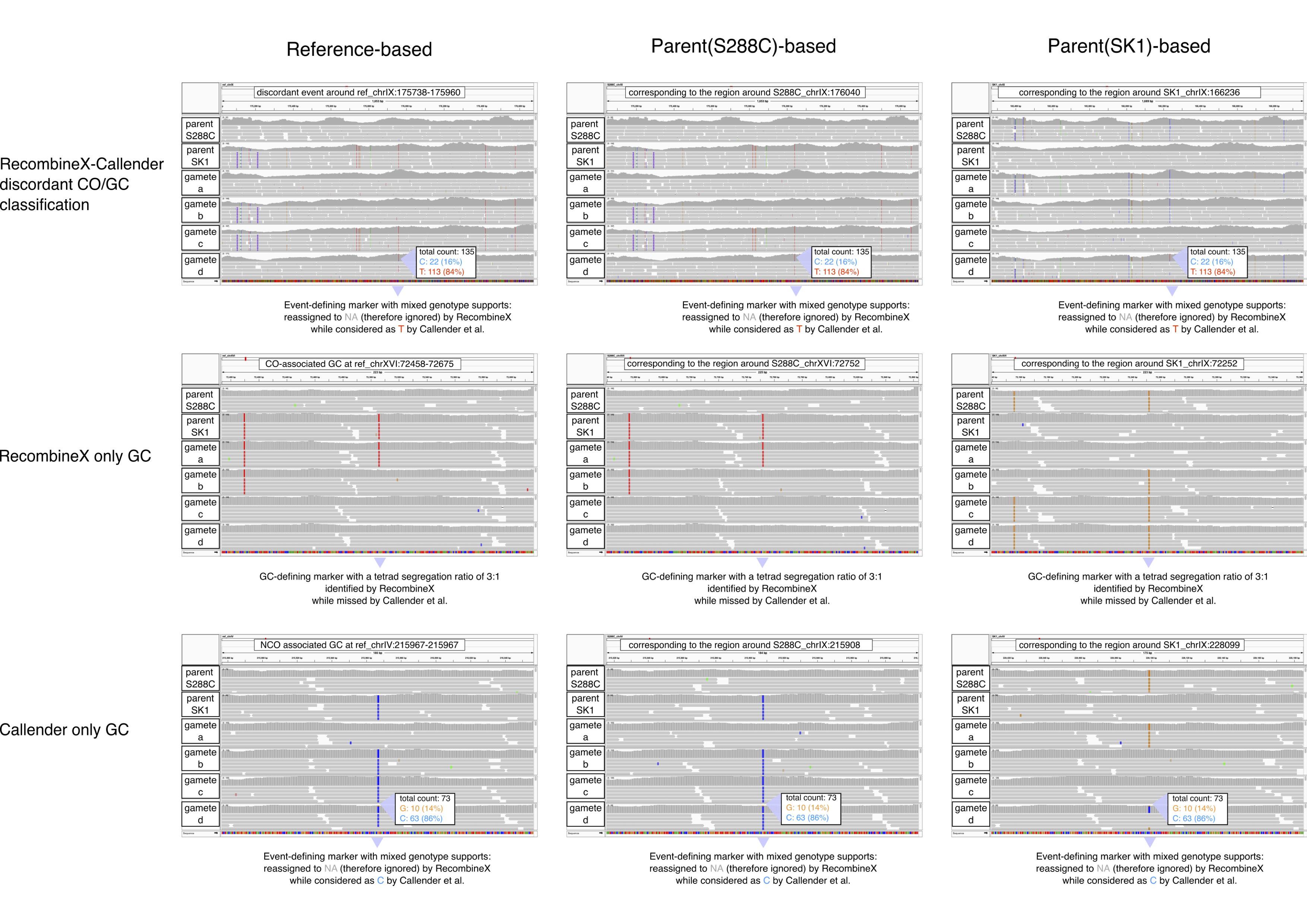

Supplement: S7 Fig — The read alignments of parent and gamete reads are visualized in IGV with event-defining SNP markers shown in colors. (TIF) [file pgen.1010047.s007.tif]

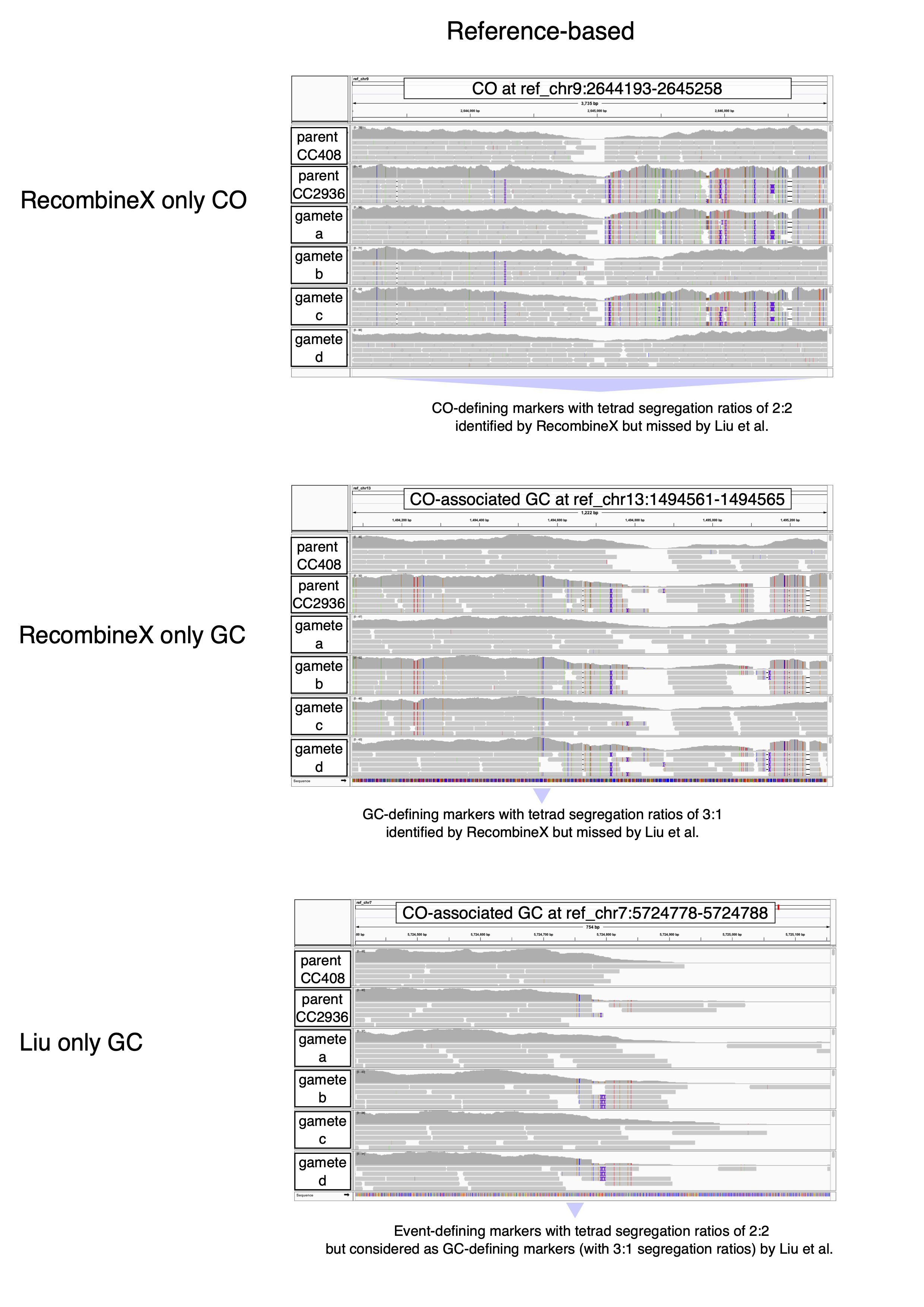

Supplement: S8 Fig — The read alignments of parent and gamete reads are visualized in IGV with event-defining SNP markers shown in colors. (TIF) [file pgen.1010047.s008.tif]
