# Supplementary material for: RecombineX: A generalized computational framework for automatic high-throughput gamete genotyping and tetrad-based recombination analysis
Source: PLoS Genet. 2022 May 9;18(5):e1010047. doi: 10.1371/journal.pgen.1010047 (PMC9119626; doi:10.1371/journal.pgen.1010047)
Supplement: Attachment — Submitted filename: RecombineX_installation_errors.pdf [file pgen.1010047.s019.pdf]

```
#####################################################################
###                                   ###
###          Welcome to RecombineX          ###
###                                   ###
#####################################################################


[2022-03-14 11:50:59] Installation starts ...

[2022-03-14 11:50:59] Defaulting to 2 concurrent jobs when executing make. Override with
MAKE_JOBS=<NUM>


[2022-03-14 11:50:59] Detected previously generated build directory.

[2022-03-14 11:50:59] Download and install all the dependencies ...

[2022-03-14 11:50:59] Installing Perl modules ...
remove previously failed installation in build//Applications/RecombineX/build/cpanm
--2022-03-14 11:50:59--  https://cpanmin.us/
Resolving cpanmin.us (cpanmin.us)... 151.101.194.217, 151.101.66.217, 151.101.2.217, ...
Connecting to cpanmin.us (cpanmin.us)|151.101.194.217|:443... connected.
HTTP request sent, awaiting response... 200 OK
Length: 302543 (295K) [text/plain]
Saving to: 'STDOUT'

-
100%[======================================================================
===================================>] 295.45K  --.-KB/s    in 0.004s

2022-03-14 11:50:59 (67.2 MB/s) - written to stdout [302543/302543]

--> Working on Test::More
Fetching http://backpan.perl.org/authors/id/E/EX/EXODIST/Test-Simple-1.302086.tar.gz ...
OK
Configuring Test-Simple-1.302086 ... OK
Building and testing Test-Simple-1.302086 ... OK
Successfully installed Test-Simple-1.302086 (downgraded from 1.302162)
1 distribution installed
Text::Soundex is up to date. (3.05)
Env is up to date. (1.04)
--> Working on Statistics::Descriptive
Fetching http://backpan.perl.org/authors/id/S/SH/SHLOMIF/Statistics-Descriptive-
3.0612.tar.gz ... OK
Configuring Statistics-Descriptive-3.0612 ... OK
Building and testing Statistics-Descriptive-3.0612 ... OK
```

Successfully installed Statistics-Descriptive-3.0612
1 distribution installed
--> Working on Statistics::Descriptive::Discrete
Fetching http://backpan.perl.org/authors/id/R/RH/RHETTBULL/Statistics-Descriptive-Discrete-0.07.tar.gz ... OK
Configuring Statistics-Descriptive-Discrete-0.07 ... OK
Building and testing Statistics-Descriptive-Discrete-0.07 ... OK
Successfully installed Statistics-Descriptive-Discrete-0.07
1 distribution installed
--> Working on Math::Random
Fetching http://www.cpan.org/authors/id/G/GR/GROMMEL/Math-Random-0.72.tar.gz ... OK
Configuring Math-Random-0.72 ... OK
Building and testing Math-Random-0.72 ... FAIL
! Installing Math::Random failed. See /Users/myname/.cpanm/work/1647258699.47395/build.log for details. Retry with --force to force install it.

###

Building BEDTools:
===
 * Creating BamTools API
DETECTED_VERSION = v2.27.1
CURRENT_VERSION =
Updating version file.
- Building in src/utils/FileRecordTools
- Building in src/utils/FileRecordTools/FileReaders
  * compiling FileRecordMgr.cpp
  * compiling FileReader.cpp
In file included from FileReader.cpp:1:
In file included from /Applications/Xcode.app/Contents/Developer/Platforms/MacOSX.platform/Developer/SDKs/MacOSX.sdk/usr/include/c++/v1/iostream:37:
In file included from /Applications/Xcode.app/Contents/Developer/Platforms/MacOSX.platform/Developer/SDKs/MacOSX.sdk/usr/include/c++/v1/ios:215:
In file included from /Applications/Xcode.app/Contents/Developer/Platforms/MacOSX.platform/Developer/SDKs/MacOSX.sdk/usr/include/c++/v1/__locale:15:
In file included from /Applications/Xcode.app/Contents/Developer/Platforms/MacOSX.platform/Developer/SDKs/MacOSX.sdk/usr/include/c++/v1/string:511:
In file included from /Applications/Xcode.app/Contents/Developer/Platforms/MacOSX.platform/Developer/SDKs/MacOSX.sdk/usr/include/c++/v1/string_view:179:

In file included from
/Applications/Xcode.app/Contents/Developer/Platforms/MacOSX.platform/Developer/SDKs
/MacOSX.sdk/usr/include/c++/v1/__string:57:
In file included from
/Applications/Xcode.app/Contents/Developer/Platforms/MacOSX.platform/Developer/SDKs
/MacOSX.sdk/usr/include/c++/v1/algorithm:649:
In file included from
/Applications/Xcode.app/Contents/Developer/Platforms/MacOSX.platform/Developer/SDKs
/MacOSX.sdk/usr/include/c++/v1/initializer_list:46:
In file included from
/Applications/Xcode.app/Contents/Developer/Platforms/MacOSX.platform/Developer/SDKs
/MacOSX.sdk/usr/include/c++/v1/cstddef:37:
../../../utils//gzstream/version:1:1: error: expected unqualified-id
1.5 (08 Jan 2003)
^

###

In file included from FileReader.cpp:1:
In file included from
/Applications/Xcode.app/Contents/Developer/Platforms/MacOSX.platform/Developer/SDKs
/MacOSX.sdk/usr/include/c++/v1/iostream:39:
In file included from
/Applications/Xcode.app/Contents/Developer/Platforms/MacOSX.platform/Developer/SDKs
/MacOSX.sdk/usr/include/c++/v1/istream:163:
In file included from
/Applications/Xcode.app/Contents/Developer/Platforms/MacOSX.platform/Developer/SDKs
/MacOSX.sdk/usr/include/c++/v1/ostream:142:
../../../utils//gzstream/version:1:1: error: expected unqualified-id
1.5 (08 Jan 2003)
^
fatal error: too many errors emitted, stopping now [-ferror-limit=]
20 errors generated.

###

In file included from SingleLineDelimTextFileReader.cpp:1:
In file included from ./SingleLineDelimTextFileReader.h:11:
In file included from
/Applications/Xcode.app/Contents/Developer/Platforms/MacOSX.platform/Developer/SDKs
/MacOSX.sdk/usr/include/c++/v1/algorithm:653:
In file included from
/Applications/Xcode.app/Contents/Developer/Platforms/MacOSX.platform/Developer/SDKs
/MacOSX.sdk/usr/include/c++/v1/memory:687:
/Applications/Xcode.app/Contents/Developer/Platforms/MacOSX.platform/Developer/SDKs
/MacOSX.sdk/usr/include/c++/v1/atomic:1055:73: error: unknown type name 'ptrdiff_t'

```
_Tp* __cxx_atomic_fetch_add(__cxx_atomic_base_impl<_Tp*> volatile* __a, ptrdiff_t
__delta, memory_order __order) _NOEXCEPT {
                                                                ^
/Applications/Xcode.app/Contents/Developer/Platforms/MacOSX.platform/Developer/SDKs
/MacOSX.sdk/usr/include/c++/v1/atomic:1060:65: error: unknown type name 'ptrdiff_t'
_Tp* __cxx_atomic_fetch_add(__cxx_atomic_base_impl<_Tp*> * __a, ptrdiff_t __delta,
memory_order __order) _NOEXCEPT {
                                                                ^
fatal error: too many errors emitted, stopping now [-ferror-limit=]
20 errors generated.
make[2]: *** [../../../../obj//SingleLineDelimTextFileReader.o] Error 1
make[1]: *** [FileReaders] Error 2
make[1]: *** Waiting for unfinished jobs....
  * compiling EmptyRecord.cpp
  * compiling Bed3Interval.cpp
  * compiling Bed4Interval.cpp
  * compiling BedGraphInterval.cpp
  * compiling Bed5Interval.cpp
  * compiling Bed6Interval.cpp
  * compiling PlusFields.cpp
  * compiling BedPlusInterval.cpp
  * compiling Bed12Interval.cpp
  * compiling BamRecord.cpp
  * compiling GffRecord.cpp
  * compiling GffPlusRecord.cpp
  * compiling VcfRecord.cpp
  * compiling NoPosPlusRecord.cpp
  * compiling BlockMgr.cpp
  * compiling StrandQueue.cpp
  * compiling RecordMgr.cpp
  * compiling RecordList.cpp
  * compiling RecordKeyList.cpp
  * compiling RecordKeyVector.cpp
make: *** [src/utils/FileRecordTools] Error 2
```

###

No env.sh file was created
