# Supplementary material for: RecombineX: A generalized computational framework for automatic high-throughput gamete genotyping and tetrad-based recombination analysis
Source: PLoS Genet. 2022 May 9;18(5):e1010047. doi: 10.1371/journal.pgen.1010047 (PMC9119626; doi:10.1371/journal.pgen.1010047)
Supplement: Attachment — Submitted filename: ResponseLetter.docx [file pgen.1010047.s020.docx]

**Response letter**

We would like to thank the three reviewers for their constructive feedback. We have thoroughly revised the manuscript as well as the associated figures and tables accordingly. In addition, a few minor modifications have been applied to the software (<https://github.com/yjx1217/RecombineX/commit/e482586b00085b763b2a231f6a2d44036e1fc45d>) accordingly. A point-by-point response to the reviewers’ comments (“replies” [R]) and a detailed description of the corresponding changes made to the manuscript (“actions” [A]) are reported below.

Comments to the Authors:

Please note here if the review is uploaded as an attachment.

**Reviewer #1:** This is an interesting methods paper describing a program called RecombineX that can be used to perform tetrad analysis from whole genome sequencing data. There are a number of interesting features, including a module for inferring the genotype of a missing 4th member of tetrad that is only represented by three sequences.

The program is admirably already publicly available. I was able to install RecombineX with some minor issues. There were multiple dependencies required for installation that were not installed in the "install_dependencies.sh" install script. For the most part these were easy to manually install and restart the script. However, the script was also unable to install DNAcopy on my system or to detect my manual installation of DNAcopy despite a reasonable amount of effort on my end- I had to edit out the command in the install script in order to complete installation.

Once that was done I was able to follow the manual to run the sample data using parental sequence data (modules 00-05). These performed as expected (except for a typo in the manual described below).

[R] We thank the reviewer for the positive feedback and helpful issue report. We fully acknowledge that the potential installation barrier of RecombineX due to its dependencies. As a preventive manner, we wrote an automatic installer bash script for RecombineX and tested it on multiple Linux server environments in both China and France. However, due to the complexity nature of computing environments, there might still be rough edges that could cause additional issues in certain situation as the reviewer encountered. Especially, we thank the reviewer for providing us more information regarding this issue, which helped us to diagnose the issue-causing problem. In this revision, we applied the following patch (with newly added parameter settings in red) to the RecombineX installation script which should fix the problem. As described below, we applied a fix which should be able to solve this reported issue.

[A] In this revision, we applied the following patch (with newly added parameter settings in red) to the RecombineX installation script “install_dependencies.sh” which should be able to fix the reported problem.

```
if [ -z $(check_installed "$rlib_dir/DNAcopy") ]; then
    clean "$rlib_dir/DNAcopy"
    if [ $(tidy_version "$R_VERSION") -ge $(tidy_version "3.6.0") ]
    then
        echo "R_VERSION=$R_VERSION, use the new bioconductor installation protocol"
        R -e ".libPaths(\"$build_dir/R_libs/\");install.packages(\"BiocManager\", repos=\"http://cran.rstudio.com/\", lib=\"$build_dir/R_libs/\");BiocManager::install(\"DNAcopy\", lib=\"$build_dir/R_libs/\")"
    else
        echo "R_VERSION=$R_VERSION, use the old bioconductor installation protocol"
        R -e ".libPaths(\"$build_dir/R_libs/\");source(\"https://bioconductor.org/biocLite.R\");biocLite(\"DNAcopy\", lib=\"$build_dir/R_libs/\", type = \"source\")"
    fi
    note_installed "$rlib_dir/DNAcopy"
fi
```

Generally, I have two main complaints about the application:

1) Use of multiple sequential modules that are not linked by a single pipeline script

2) Running the modules requires manually editing individual script files

I would encourage the authors to write a pipeline script with reasonable default values that allows a user to run the program from the command line interface without substantial manual building of files. This would lower the barrier to others using RecombineX in their own work.

[R] We thank the reviewer for valuable suggestions, and we acknowledge the advantages that such modification could bring in terms of ease of use. We chose the current design structure mainly based on following considerations: 1) Since a few computationally intensive (and therefore time-consuming) steps (e.g., read mapping) were involved, we think it might be helpful to divide the entire computational workflow into multiple separate modules so that users could perform their analysis in a module-by-module manner, which should help in terms of robustness and flexibility. Also, such semi-independent and editable design of different modules makes it convenient for users to customize and combine different modules for suiting their specific workflow. This been said, we are happy to keep exploring more software packaging choices to strike a better balance between ease of use and ease of adaptation in future, maybe with the help from professional software engineers.

All that said, the paper itself is in pretty good shape and does a pretty satisfying job of demonstrating the capabilities of RecombineX, including identifying CNVs and structural changes missed by previous approaches and validating them with nanopore sequencing. I would encourage the authors to make several of their figures much larger. They are difficult to read as is.

[R] We are grateful for reviewer’s kind words, and we apologize for the small figures. In the revised manuscript, we reorganized and refined those figures to improve their readability (see the responses below for details).

Specific Comments:

Figure 1 "Gamete Reads" part in upper right is a little misleading. This suggests every paired end read is a fusion of parent 1 and parent 2. Pretty sure this isn't as intended.

[R] The reviewer is correct. Our use of the red-blue color combination for denoting gamete paired-end reads is merely to reflect the fact that those gametes inherited the genomic backgrounds of both parents (denoted in red and blue respectively). We now see how it can be misleading and have changed the color of gamete reads in the revised manuscript to avoid such confusion.

[A] The color of gamete reads in Figure 1 has been changed into light purple.

Page 4, Wang et al. 2012 is double cited "Wang et al. 2012, 2012."

[R & A] We apologize for this omit and has corrected in the revised manuscript.

Figure 4 A-D are unreadably small on the page. By zooming in, you can also see there are some labels that are partially covered in white boxes.

[R & A] We divided this figure into two separate figures (new Fig 3 and Fig 4 respectively) so that the graphic and texts of this figure could be better read. Also, the graph labels were further adjusted.

Figure 6 is very small.

[R & A] We enlarged the text labels in this figure (new Fig 6) to improve its readability.

Figure 7 again is unreadably small.

[R & A] We divided this figure into two separate ones (with the original Figure 7A-7C as new Fig 7 and the original Figure 7D-7E as new Fig 8) to improve the readability.

Error in the manual for the sample data:

cd ./../ RecombineX.03.Gamete_Read_Mapping_to_Reference_Genome.sh

bash RecombineX.03.Gamete_Read_Mapping_to_Reference_Genome.sh

The RecombineX. should be deleted from the first of those commands.

[R & A] Apologize for this omit and it has been corrected in the revised manual (page 17 in the updated manual file: Manual_20220403.docx).

**Reviewer #2:** The authors developed a software package to analyze meiotic recombination along the genome from sequencing data of the four meiotic products (tetrad analysis). The pipeline can be employed to perform tetrad analysis in any organism in which it is possible to sequence the genomes of the four products resulting from a single meiosis. The package includes several modules that allow performing all the different steps of the analysis required for tetrad analysis when starting from raw genomic sequencing reads: genotyping of the parents to identify polymorphic markers, genotyping the meiotic products and identification of the recombination events from the genotypes. Marker identification can be performed based on a reference genome of the species in question or directly from sequencing data of the specific parental lines used for the cross. The software also includes a module to simulate the genotypes of the meiotic products that can be used to test deviations from empirical data. After testing the package analyzing simulated genotypes, the authors assess its performance with tetrad data from budding yeast and a green alga, showing that the resulting recombination profiles are very similar to those determined in the original works. As the authors put it, the main advantages of the software are that all the steps needed for tetrad analysis can be performed using a single package, and the fact that it can be employed for any organism where tetrad analysis can be performed.

Below are my specific comments ordered from mayor to minor.

1. Can the package deal with diploid parental genomes? Several strains and organisms have been sequenced in their diploid state and their genome assemblies are not phased (PMID: 35210580, for example). It would be important to discuss the potential/limitations of the software in that respect.

[R] In 02.Polymorphic_Markers_by_Reference_based_Read_Mapping and 13.Polymorphic_Markers_by_Cross_Parent_Read_Mapping modules, RecombineX only consider homozygous SNVs as potential parental markers, regardless of the ploidy status of the parental genomes. So RecombineX will work with both haploid and diploid parents by design.

[A] We clarified this point in the revised manuscript (page 7 in the file RecombineX_Manuscript_PLOS_Genetics_R1_tidy.docx):

*“Both haploid and diploid parent genomes are supported, and in both cases only homozygous SNVs will be considered. Such design choice helps avoiding the risk of including ambiguous markers for most use scenarios, although understandably limits the application of RecombineX on diploid parental genomes with very high heterozygosity.”*

Similar explanation has also been added to the updated manual (page 6 in the file Manual_20220403.docx):

*“Please note that the actual crossing parents can be either haploids or diploids, and only homozygous SNVs will be considered for parental markers. Such design choice helps avoiding the risk of including ambiguous markers for most use scenarios, although understandably limits the application of RecombineX on diploid parental genomes with very high heterozygosity.”*

2. The two modes for identifying markers could be better explained. For example, in the parent-based mode it is not clear whether genome assemblies of the parents are a prerequisite or whether only having sequencing reads of the parents is enough. This is also confusing when the authors perform the analysis in the green alga since they mentioned that they only used the reference mode given that no parental assembly was available. In the reference mode the reference assembly is needed and reads of one parent, or the assembly of one parent? Please clarify the requirements.

[R] We apologize for lack of clarity. For the reference-based mode, a reference genome assembly, and the short reads from the two crossing parents are needed for marker identification. For the parent-based mode, the native genome assemblies of the two crossing parents are required at the minimal for marker identification. And the short reads from the two crossing parents could be further incorporated for more stringent marker identification.

[A] We clarified this point in the revised manuscript.

On the page 5-6 of the file RecombineX_Manuscript_PLOS_Genetics_R1_tidy.docx we added:

*“The reference-based mode requires a reference genome assembly as well as the short reads of the two crossing parents. The parent-based mode requires the native genome assemblies of both crossing parents, while the short reads of the two crossing parents can be further used when available (S1 Fig). Both reference-based mode and parent-based mode further need short reads of individual gametes, ideally labeled based on their original tetrad contexts.”*

And on the page 23 of the file RecombineX_Manuscript_PLOS_Genetics_R1_tidy.docx we added:

# *“Expected input data*

*Depending on the available input data, RecombineX can be executed in two modes: 1) the reference-based mode and 2) the parent-based mode. The reference-based mode requires a reference genome assembly as well as the short reads of the two crossing parents. The parent-based mode requires the native genome assemblies of both crossing parents, while the short reads of the two crossing parents can be further used when available. Please note that the actual crossing parents can be either haploids or diploids, and in latter case, only homozygous SNVs will be considered for parental markers for downstream analysis. In both modes, short reads of individual gamete genome sequencing are further needed for gamete genotyping and recombination landscape profiling (if gametes are labeled based on their corresponding tetrad context).”*

The same information has also been added to the updated manual (page 6 in the file Manual_20220403.docx).

3. Given that the package needs different software to be run it would be important to mention the computational requisites in the methods so that the feasibility of running the package is clear.

[R] We did provide a prerequisite list in RecombineX’s GitHub depository (<https://github.com/yjx1217/RecombineX/blob/master/prerequisite.txt>) but admittedly have not done so in the manuscript. These prerequisites are standard Linux compilers and libraries that are highly likely to be pre-installed on most Linux system. In addition to these prerequisites, RecombineX relies on a series of third-party bioinformatics tools for data analysis, all of which can be automatically installed and configured via a pre-shipped installation script.

[A] In the revised manuscript, we added detailed description of these prerequisites and dependencies in the Materials and Methods section (page 22-23 in the file RecombineX_Manuscript_PLOS_Genetics_R1_tidy.docx and the S8 Table in the file Supplementary_Tables_R1.xlsx). Similar information has also been added to the updated manual (page 4-5 in the file Manual_20220403.docx):

4. When mentioning that almost all previously identified events in the yeast example were recovered it may be better to give exact percentage or number of recovered events. Similarly when mentioning events only found by RecombineX, and throughout, when comparisons with previous studies are done. For example, in Figure 6 it seems there are more events only identified by Callender et al., it would be better to include the actual numbers in the text. It may be also important to mention very briefly how previous events were identified to understand the possible reasons for the discrepancies.

[R & A] We added the detailed numbers and percentages to the main text for describing these results (on the page 16 in the file RecombineX_Manuscript_PLOS_Genetics_R1_tidy.docx for these changes):

*“In sum, RecombineX recovered 95.4% (378/396) of previous reported CO events and 89.9% (456/507) of previous reported GC events (Fig 6A and S6 Table)”*

As for the cause of the discrepancies, we think the reason is that the original study didn’t explicitly apply stringent filters on repetitive regions, CNVs, and ambiguous genotypes. In the revised manuscript, we added this explanation to the main text (also on the page 16 in the file RecombineX_Manuscript_PLOS_Genetics_R1_tidy.docx):

*“As for those only called by the original study (18 COs and 51 GCs), we found they were mostly filtered out by RecombineX at either marker identification or genotyping stage due to repeat/CNV-association or ambiguous genotypes. Therefore, the comparatively more events that were only identified by the original study are likely explained by the lack of explicit and stringent quality-control filters to account for repetitive regions, CNVs, and ambiguous genotypes during marker identification and gamete genotyping”.*

5. Figure 4E and F it is not clear why lower Qnet cutoffs would give higher number of correct genotypes.

[R] By design, RecombineX employs a series of quality control filters (e.g., repetitive-region filter, CNV-region filter, genotyping purity filter, and biparental reciprocality filter) to minimize the chance of genotyping errors. Therefore, even with a low Q_net_ (e.g., 10), the chance of assigning false genotyping is still quite low. The apparent performance gain with lower Q_net_ cutoffs revealed in the original Figure 4E and 4F (corresponding to the new Figs 4A and 4B in the revised manuscript file RecombineX_Manuscript_PLOS_Genetics_R1_tidy.docx), especially when sequencing depth is low, is largely due to the impact of sequencing depth. The way that we calculate Q_net_ determines that its quantity is partially linked with sequencing depth. When sequencing depth is very low (e.g., 1X or 2X) and our Q_net_ cutoff is very high (e.g., 100), it will be impossible for most markers to pass our Q_net_ cutoff simply because not enough reads were mapped to that marker position, leaving those markers genotyped as “NA” and thus considered as with missing genotypes. In our original manuscript, we sort of touched on this but maybe not explicit enough:

“Aside from sequencing depth, Qnet cutoff is another important parameter for RecombineX’s genotyping analysis, which specifies the cumulative genotyping-supporting score leveraged over all mapped reads at a given marker site. While a higher Qnet cutoff helps to filter out ambiguous genotyping signals, setting it too high will compromise RecombineX’s genotyping power for shallowly sequenced tetrads, leaving the genotypes of many markers as undetermined (Figure 4E and Table S2).”

[A] In the revised manuscript, we added more explanations to clarify this point (on the page 11 in the revised manuscript file RecombineX_Manuscript_PLOS_Genetics_R1_tidy.docx):

*“Aside from sequencing depth, Q_net_ cutoff is another important parameter for RecombineX’s genotyping analysis, which specifies the cumulative genotyping-supporting score leveraged over all mapped reads at a given marker site. A higher Q_net_ cutoff helps to filter out ambiguous genotyping signals and thus ensures better genotyping accuracy. But it is also important to point out that the value of Q_net_ roughly scales with sequencing depths, therefore setting it too high will compromise RecombineX’s genotyping power for shallowly sequenced tetrads, leaving the genotypes of many markers as undetermined (Fig 4B and S2 Table). Luckily, since RecombineX employs a series of quality control filters (e.g., repetitive-region filter, CNV-region filter, genotyping purity filter, and biparental reciprocality filter) to minimize the chance of marker identification and genotyping errors, even with a lenient Q_net_ cutoff (e.g., 10), the chance of assigning false genotypes is still reasonably low. Therefore, in general, lenient Q_net_ cutoffs such as 10 or 20 are recommended for tetrad with very shallow sequencing depth (e.g., 1X). In this case, the performance gain from getting more markers genotyped substantially outweighs the theoretical higher risk of getting erroneous genotypes, which is demonstrated by our simulation.”*

6. The author mentioned that it is possible that changing the default parameters of RecombineX it may be possible to increase the concordance with past results. Have they actually attempted trying different parameters with the real yeast and alga data?

[R] As pointed out in several previous studies (Wijnker et al. eLife, 2013; Qi et al. PNAS 2014), marker identification and genotyping analysis could be substantially compromised by confounding factors such as copy number variation and erroneous mapping, and therefore inflate the number of CO and GC events identified. During our development of RecombineX, we also noticed such issue when testing it with both simulated and real dataset without additional filters. This issue is more severe for GC events. Therefore, we introduced several quality-control filters/parameters in RecombineX to prevent such risk, and as mentioned in our manuscript, we think these filters/parameter settings are “generally beneficial for preventing the inclusion of suspicious markers potentially derived from unreliable read mapping”. It worth mentioning that when testing and making decision of these filters/parameters, we relied on manual IGV-inspection as our gold standard. The result consistency with the original studies has not been taken into consideration during this process.

[A] We rephrased those sentences for comparing our re-analysis with the original studies to avoid leaving the impression that “it is possible that changing the default parameters of RecombineX may be possible to increase the concordance with past results” as the reviewer pointed out. See the page 17 in the revised manuscript file RecombineX_Manuscript_PLOS_Genetics_R1_tidy.docx for this change:

*“As for discrepant GC calls, our repetitive/CNV-region check and manual IGV inspection suggests that most of them are likely explained by the comparatively less stringent marker and genotype filtering of the original study, especially in complex genomic regions (S8 Fig).”*

7. In Figure 5 it would be important to clarify what are incorrect events (middle panels). Especially because the tendencies in the graphs of correct and missing events seem to be exact reciprocal of each other, leaving no room for incorrect events.

[R] We apologize for the lack of explanation here in the original manuscript. These “incorrect events” are false positive events that were not simulated at all. Such incorrect event calling could occur when our simulated event-associating markers are with missing genotypes, which together with their nearby markers that were correctly genotyped created apparent genotype patterns that deviated substantially from the simulated ground truth. This explains why all incorrect event calling occurred when Q_net_ cutoff is high and sequencing depth is low.

[A] We add this explanation to the corresponding paragraph (on the page 16 in the revised manuscript file RecombineX_Manuscript_PLOS_Genetics_R1_tidy.docx):

*“Especially, with high Qnet cutoff but low sequencing depth, we observed some false positive events (referred as “incorrect events” in Fig 5) that were not simulated. These events were called due to the fact that some simulated event-associating markers were genotyped as “NA”, leaving their nearby markers creating apparent event-supporting genotype patterns substantially deviated from the truth, which eventually leads to incorrect recombination event calls.”*

8. In Figure 4, the genotypes of tetrads in panels A to D are too small, it is not possible to see the exact genotypes. Either enlarge the figure or only show some of the chromosomes, but larger so that the genotypes can be distinguished. In panels E and F, 10 Qnet cutoffs were tested, but only a couple of lines can be seen in the figure, always less than 10. Also, from the color legend of the Qnet cutoffs it would seem that the cutoffs tested are a continuous, but they are rather 10 discrete cutoffs (same in Figure 5).

[R & A] We apologize for these figure related issues and have re-arranged/refined these figures in the revised manuscript to improve their readability and avoid potential confusion. Please see the new Fig 4 and Fig 5 for the corresponding changes. The reason why there are <10 lines in the figure is because those lines partially overlapped with each other quite often and thus become less visible.

9. Figure 7 has the same problem as the upper panels of Figure 4, the tetrad genotypes are too small to see the recombination events. In general, in this figure there is a lot of information in a small space making it hard to understand.

[R & A] We acknowledge the dense information packed into this figure might be too overwhelming and have divided this figure into two separate figures (new Fig 7 and Fig 8) to hopefully improve the readability.

10. In Figure 3 it is not clear what is meant by “markers whole genome alignment” and why the line is steady unlike the reference- and parent-based markers.

[R] “markers by whole genome alignment” refers to markers identified by module 12, in which the native genome assemblies of the two crossing parents were align to each other for marker identification. This marker set only depends on the parental genome assemblies and should still be considered as parent-based marker set, which can be directly used for downstream genotyping and recombination events profiling (as the dashed arrow indicates in the new S1 Fig). When users are also in process of parental reads (which is usually the case), module 13 and module 14 can be further applied to refine the whole-genome-alignment markers to form a more refined parent-based marker set.

[A] In the revised manuscript, we also rephrased corresponding sentences describing this result to improve the clarity (on the page 9 in the revised manuscript file RecombineX_Manuscript_PLOS_Genetics_R1_tidy.docx):

*“In parent-based mode, RecombineX identified 49,669 markers based on parental genome alignment alone (referred as markers identified by whole-genome-alignment). When parental reads were further supplied for reciprocal parent read mapping and consensus marker identification, the corresponding consensus marker count scales with the sequencing depth of parent reads.”*

11. I am not sure Figure 2 should be a main figure since it is a rather technical representation of the software that does not bring much to the reader unless they are direct users of the package.

[R & A] In the revised manuscript, we moved this figure to supplementary figures (as the new S1 Fig) following the reviewer’s suggestion.

12. Apart from the organisms that the authors mention where tetrad analysis can be performed, there have also been efforts to do them in animals (PMID: 25151354). Although these experiments are currently technically very challenging, they may become more conventional in a near future. It may be worth mentioning them as a possible future use of the software.

[R & A] We thank the reviewer for this insightful suggestion. In the revised manuscript, we added this point in the final paragraph of the manuscript (on the page 22 in the revised manuscript file RecombineX_Manuscript_PLOS_Genetics_R1_tidy.docx):

*“Finally, efforts have been made to perform tetrad analysis for mammals (Cole et al. 2014). Such experiment, although remains technically challenging, might become conventional in near future along with the rapid advancement of single-cell sequencing. Therefore, we expect RecombineX to empowers future tetrad analysis for extended species”.*

13. Would be good to add reference PMID: 21481229 when referring to PMS.

[R & A] We added this reference into the introduction section (on the page 7 in the revised manuscript file RecombineX_Manuscript_PLOS_Genetics_R1_tidy.docx).

14. The reference to IGV should be used the first time IGV is mentioned and “IGV browser” may be clearer, at least for the first instance, than just IGV.

[R & A] We moved the reference for IGV to the location where it first appears in the manuscript (on the page 16 in the revised manuscript file RecombineX_Manuscript_PLOS_Genetics_R1_tidy.docx).

15. Some of the colors of lines in Figure 3 are difficult to distinguish (blue, purple and green).

[R & A] We modified the color scheme used in this figure (the new Fig 2) to improve its readability.

16. In the sentence “Moreover, additional tools are available for reconstructing the gamete-tetrad correspondence map from random gametes (Sakhanenko et al. 2019),…” it would be important to mention that these are previously developed tools. The start of the sentence misleads the reader to think that the tools are part of RecombineX.

[R & A] We rephrased this sentence to avoid potential confusion (on the page 10-11 in the revised manuscript file RecombineX_Manuscript_PLOS_Genetics_R1_tidy.docx):

*“Moreover, additional tools have been previously developed for reconstructing the gamete-tetrad correspondence map from random gametes (Sakhanenko et al. 2019), which could be used in combination with RecombineX when processing sequencing data from randomly collected gametes.”*

17. In the legend of figure 1B instead of saying “Tetrad genotyping” it may be better to say “Gamete genotyping” as it is used in the figure itself.

[R & A] We corrected this figure legend following the reviewer’s suggestion.

18. In the sentence “…the sampled tetrads are derived from a cross between S288C and YPS128 strains,…” it may be better to use SK1 instead of YPS128 as it is used in the rest of the article.

[R & A] We apologize for the omit. Yes, it should be “SK1” rather than “YPS128”. We corrected this error in the revised manuscript.

19. Check typos and syntax in the following sentences:

• have substantially expand our

• inspecting individual meiosis event, which

• generation of computational solution for high-throughput

• for polymorphic markers identification, gametes genotyping

• Upon the obtaining of parental markers

• making this assumption holds in general.

• inference, RecombineX’s raw genotyping function do not require any tetrad information as the priori when performing raw genotyping.

• original study, with RecombineX that completely recovered

• Type 1 and Type 2 GC respectively

• which could make significant impacts on the genotypes

[R & A] We apologize for these typos and syntax errors and have tried our best to correct them in our revised manuscript.

**Reviewer #3:** Li et al describe RecombineX, a software package for processing and analyzing genetic recombination data from tetrad analysis. The manuscript is clear, well written, and the software seems useful. It is significantly more powerful than the ReCombine package from a different group (Anderson et al 2011, cited) which, as far as I know, is the only comparable piece of software. In particular, RecombineX can be run in a mode that does not require one of the two parents to be the “reference” strain of the species (this is a substantial limitation of the older ReCombine package). Li et al also tested RecombineX extensively by simulation, allowing them to conclude that it performs very well and that it can even detect most recombination events when sequencing coverage of the gametes is very low, about 1-2x.

RecombineX will be a useful tool. It might be argued that the manuscript would be a better fit to PLOS Computational Biology, but in my view the audience for this software is geneticists, so the manuscript is appropriate for PLOS Genetics.

My main comment is that I ran into difficulties when I tried to install RecombineX on my computer (Macbook Pro laptop), so I was unable to actually run the program myself. I will attach some parts of the log file as an appendix to this review in the hope that the authors can troubleshoot it. This is not a major criticism. The authors have provided extensive documentation on GitHub to support the software, and I know that it is extremely difficult to anticipate all the installation problems and software dependencies that users will encounter. In my case, the installation script stopped running during the section “Installing Perl modules” because installation of the Math::Random library failed. When I commented out this line in the install_dependencies.sh script, I then got multiple errors during the attempted installation of BedTools. In the end, no env.sh file was created so I was not able to run RecombineX at all.

[R] We thank the reviewer’s understanding of the complexity involved in bioinformatics software development. As mentioned in our reply to reviewer 1, we made our efforts to provide user an installation script for automatically install and configure RecombineX, which hopefully can help to eliminate the installation barrier. As for those errors that reviewer 3 encountered, we think the problem is highly likely triggered by the system-level differences between Linux and MacOSX. RecombineX is designed and tested for x86-64-bit Linux operating system. While MacOSX is quite similar to Linux on surface, there are still some important differences underneath, which may impact the compilation of some third-party tools (just like the errors that the reviewer encountered). Therefore, some system-specific modification will be needed to adapt RecombineX for Mac OSX.

I would like to suggest: (1) The authors should try troubleshooting the installation on a few different computer systems. (2) At the moment it looks like RecombineX will not run at all unless every other program that it depends on is installed successfully and correctly. Consider providing an option for users to do a minimal installation, in which RecombineX could start running even if some of its functions won’t work. For example, I can see that it installs the NCBI BLAST package, but I think that this is only required for the non-reference genome mode, so it should be possible to run RecombineX in reference-genome mode even if BLAST is not installed. It would also be useful to provide documentation explaining which pieces of external software are required for each function of RecombineX, so that a user could install only the pieces of external software that they will actually need.

[R & A] We thank the reviewer’s valuable suggestions. During the development of RecombineX, we tested it on multiple Linux-running computing servers in China and France. Therefore, we explicitly described RecombineX as a Linux-based computational framework in our manuscript and the GitHub depository. For Linux-based system, the installation should be relatively straightforward. An extend support for Mac OSX is definitely worth considering and exploring in near future. Alternatively, we are also considering the possibility of packaging and distributing RecombineX via a system-independent channel such as bioconda, but it will require substantially more efforts and time. As for the reviewer’s suggestion on better explaining the function that each dependency undertakes, in the revised manuscript (and RecombineX’s manual) we added a detailed supplementary table (the new S8 Table) to provide such information. Finally, regarding the reviewer’s suggestion on minimal installation, in the revised manual, we added a dedicated note to guide users on how to skip the installation of certain dependencies when needed (e.g., when there is already an existing installation of the corresponding dependency in the system) (see page 9 in the updated manual file Manual_20220403.docx).

The structural rearrangement in Figure 7C is unusual and interesting, and I think that it deserves to be mentioned in the Abstract.

[R & A] We added the following sentences into the Abstract to reflect this point:

*“Interestingly, our re-analysis of the budding yeast tetrad sequencing data with RecombineX and Oxford Nanopore sequencing revealed two unusual structural rearrangement events that were not noticed before, which exemplify the occasional genome instability triggered by meiosis.”*

Page 5, end of Introduction: Please include a description of what input data is required by RecombineX. I can see that it requires Illumina reads files from 4 gametes in a tetrad, and either a reference genome assembly or assemblies of each of the 2 parents. Does it also require reads files from the 2 parents? Are any other input files required?

[R] We apologize for lack of clarity. For the reference-based mode, a reference genome assembly, and the short reads from the two crossing parents are needed for marker identification. For the parent-based mode, the native genome assemblies of the two crossing parents are required at the minimal for marker identification. And the short reads from the two crossing parents could be further incorporated for more stringent marker identification. In both modes, gamete sequencing reads are needed.

[A] We added this clarification into the revised manuscript (page 23 in the updated manual file Manual_20220403.docx).

# *“Expected input data*

*Depending on the available input data, RecombineX can be executed in two modes: 1) the reference-based mode and 2) the parent-based mode. The reference-based mode requires a reference genome assembly as well as the short reads of the two crossing parents. The parent-based mode requires the native genome assemblies of both crossing parents, while the short reads of the two crossing parents can be further used when available. Please note that the actual crossing parents can be either haploids or diploids, and in latter case, only homozygous SNVs will be considered for parental markers for downstream analysis. In both modes, short reads of individual gamete genome sequencing are further needed for gamete genotyping and recombination landscape profiling (if gametes are labeled based on their corresponding tetrad context).”*

Same information has also been added into the updated manual (on page 6 of the file Manual_20220403.docx).

Figure 4: In panels A-D, it is quite difficult to see the red and blue colors because the gray boxes around each chromosome are quite thick. I can see the colors on my screen if I zoom-in a lot on the PDF, but on a printout and onscreen at 100% magnification they are too difficult to see. This comment also applies to Fig 7A,B. Consider making the chromosome boxes thinner, or use a single line instead of a box.

[R & A] We apologize for the figure quality. In the revised manuscript, we re-organize and refine these figures to improve their readability.

For the S. cerevisiae tetrads, was the second parent in the cross strain YPS128 (page 15) or SK1 (pages 17 and 24)? And do the tetrad names begin with AND7 (Fig 6 legend), or AND17 (text on page 17) ??

[R & A] We apologize for these omits. They should be “SK1” and AND17 respectively. We corrected these errors in the revised manuscript.

(See also attached PDF file of installation errors).

[R] We thank the reviewer for providing the detailed error message which helps us to understand how the errors occurred.
